# Evaluation of the Impact of Different Natural Zeolite Treatments on the Capacity of Eliminating/Reducing Odors and Toxic Compounds

**DOI:** 10.3390/ma14133724

**Published:** 2021-07-02

**Authors:** Vanda Liliana Babalau Fuss, Gabriel Bruj, Lucian Dordai, Marius Roman, Oana Cadar, Anca Becze

**Affiliations:** 1 INCDO-INOE2000, Research Institute for Analytical Instrumentation, 67 Donath Str., 400293 Cluj-Napoca, Romania; vanda.fuss@icia.ro (V.L.B.F.); lucian.dordai@icia.ro (L.D.); marius.roman@icia.ro (M.R.); oana.cadar@icia.ro (O.C.); 2 Enviro Naturals Agro LtD., 12A Preciziei Str., 062203 Bucharest, Romania; gabriel@enviro-naturals.com

**Keywords:** natural zeolite, thermal treatment, chemical treatment, odor, polycyclic aromatic hydrocarbons

## Abstract

Unlike odorants that mask odors, natural zeolite acts as a molecular sieve that captures and eliminates odors. Different treatment methods can be applied to influence the properties of the natural zeolites. To enhance the odor adsorption capacities of the natural zeolite two types of treatment methods were applied: chemical (acid, basic) and thermal. The initial natural zeolites and the activated one were characterized using X-ray diffraction (XRD) and scanning electron microscope (SEM-EDX). Two experiments were performed to establish the odor adsorption capacity of the activated natural zeolites. The best zeolite for the adsorption of humidity, ammonia and hydrogen sulfide was the 1–3 mm zeolite activated through thermal treatment. For the adsorption of PAHs, the best zeolite was the one activated through basic treatment, with an adsorption capacity of 89.6 ng/g.

## 1. Introduction

The smell of a product is a complex, gaseous mixture that can contain hundreds of individual chemical components [1]. Malodors can have a direct impact on the health of humans and animals because they can contain harmful airborne substance [2,3]. Managing odors represents a challenge for the continuous growth of the global industrialization. For household malodor, with consumers that are more and more interested in eco-friendly solutions, optimizing the available natural resources is a simple way of ensuring odor control with little impact on the environment. Spoiled food smells are the results of bacterial decomposition of organic matter. The ability to sense these smells and recognize them as off-putting is essential in avoiding food intoxication [4,5]. People have found different ways to ensure food stability. One of them is to reduce the humidity of the environment where food was stored [6].

The persistent smell of tobacco smoke is a toxic mixture of more than 5000 compounds [7]. Polycyclic aromatic hydrocarbons (PAHs), which are the result of incomplete burning of the organic material, are some of the carcinogenic compounds that tobacco smoke contains, especially benzo(a)pyrene which is one of the most potent carcinogens [8,9]. Eliminating PAHs from the households using natural available materials will ensure a safer environment.

Strategies for odor elimination include chemical reactions, adsorption or absorption, and combinations of these approaches [10].

The natural zeolites are crystalline materials with a porous structure that can accommodate a wide variety of cations (i.e., Na^+^, K^+^, Ca^2+^, Mg^2+^, etc.) [9,11]. Zeolites have superficial interaction properties with changeable organic molecules that have a positive charge or can absorb polar molecules [12]. Due to their unique structural and chemical composition, such as high adsorption and condensation properties, high surface area, and adjustable surface property [13,14], zeolites are promising candidates for adsorbents applications [15]. Several studies of biofilter technology for treating a variety of odors emissions have been reported [16,17,18,19]. Odor gases are removed by processes thought to include adsorption/absorption and bio-oxidation in the biofilter media [20]. Biofilters are an efficient and practical technology for gas cleaning and can reduce odors to acceptable levels [20]. In previous studies [20,21,22], hydrogen sulfide, H_2_S, and ammonia (NH_3_) were effectively eliminated with a removal efficiency of over 90% with different types of biofilters.

These properties allow them to be applied into different fields, including gas purification and wastewater treatment. Zeolite is an aluminosilicate-type microporous material with three-dimensional tetrahedral SiO_4_ and AlO_4_ units. Each AlO_4_ unit introduces a net negative charge and requires to be constructed by extra exchangeable cations [23]. Then the cations are held loosely and can easily be exchanged with others. This connectivity of these tetrahedral SiO_4_ and AlO_4_ units determine the framework type of zeolites [24].

The mining of natural zeolites typically involves crushing, milling, and grinding techniques [25]. Zeolites are usually supplied in the form of a range of mesh size. In this study, the samples have a particle size of 1–3 mm and 3–5 mm. The zeolite materials have an ordered inner channel and homogeneous pore size distribution, which can control the approach of adsorbates to their internal space [26]. Zeolites are considered a type of effective adsorbent for the removal of various pollutants due to their low production cost, high surface area, excellent thermal stability and ordered pore structure.

The aim of this research is to evaluate the capacity of the natural zeolites to adsorb odors (H_2_S, NH_3_) and toxic compounds (PAHs) from the household environment, after undergoing chemical and thermal treatments. Two experiments were set up that mimic, at laboratory scale, household areas where natural zeolites can be used for their adsorbent properties. H_2_S, NH_3_ were generated in a container using pork meat left to decomposes as source, while PAHs were obtained using tabaco smoke. Enhancing the natural zeolites capacity of adsorption can ensure a wider applicability.

## 2. Materials and Methods

### 2.1. Zeolite Samples

The natural zeolites samples, with a grain size in the range of 1–3 mm (Z-1) and 3–5 mm (Z-2), originated from Poland. They were provided by the company Enviro Naturals Agro LtD., Bucharest, Romania.

### 2.2. Materials

The refrigerated minced pork meat was bought from a local supermarket (Cluj-Napoca, Romania), with a fat content of under 20%. The tobacco used in the experiments is Silverado Blue Extra Volume tobacco, which is a voluminous tobacco, of superior quality, that was cut into strands and purchased from specialty stores. The tobacco does not contain artificial flavors and the strength is medium to strong. Hexane, acetonitrile gradient grade are from VWR (Fontenay-sous-Bois, France). Sodium hydroxide, pure pellets, from Merck(Darmstadt, Germany) and chlorhydric acid are from LGC Promochem (Wesel, Germany). PAHs Mix (naphthalene, acenaphthene, fluorene, phenanthrene, anthracene, fluoranthene, pyrene, benzo(a)anthracene, chrysene, benzo(b)fluoranthene, benzo(k)fluoranthene, benzo(a)pyrene, dibenzo(a,h)anthracene, benzo(g,h,i)perylene, indeno (1,2,3-cd)pyrene) 10 µg/mL each in acetonitrile are from Sigma Aldrich (St. Louis, MO, USA). Ultrapure water (18.2 MΩ/cm) obtained from a Millipore Direct-Q3 UV Ultrapure water system (Millipore, Molsheim, France) was used.

### 2.3. Zeolite Treatments

Two types of treatment methods for zeolite activation were applied: chemical (acid, basic) and thermal. The acid treatment was done using HCl 0.4 M for 2 h. After the acid treatment the zeolite was washed with ultrapure water until no Cl^-^ ions were detected in the washing water by using AgNO_3_ solution, and then the washed zeolite was dried at 140 °C for 2 h. The basic treatment was done using with NaOH 1 N for 2 h, while stirring, at 80 °C. After the basic treatment, the zeolite was washed with ultrapure water until the pH = 7, and then dried at 140 °C for 2 h. The thermal treatment was performed at 300 °C for 3 h (Table 1).

### 2.4. Characterization

The powder X-ray diffraction (XRD) patterns were recorded at room temperature using a D8 Advance (Bruker, Karlsruhe, Germany) diffractometer operating at 40 kV and 40 mA with CuK_α_ radiation (λ = 1.54060 Å). The degree of crystallinity was estimated from the relative intensities of the most characteristic peaks of clinoptilolite, taking as reference the intensity of these reflections in the initial zeolite sample [27]. To evaluate the composition and morphology, the zeolites were analyzed using the scanning electron microscope SEM VEGA3 SBU-EasyProbe (Tescan, Bron, Czech Republic) with energy-dispersive X-ray spectroscopy Quantax 200 EDX detector (Bruker, Berlin, Germany). The zeolite samples were mounted on the aluminum stud using a double-sided adhesive carbon tape and measured induplicate. The conversion to the corresponding oxide was made by multiplying the element concentration with 1.8895 (Al_2_O_3_), 1.4297 (Fe_2_O_3_), 1.3392 (CaO), 1.6583 (MgO), 1.2046 (K_2_O), 1.3480 (Na_2_O), while SiO_2_ and loss of ignition (LOI) were determined by a gravimetric method [27].

### 2.5. Experimental Plan 

Experiment 1:100 g of pork minced meat and 30 g of zeolite were introduced in 1 L containers with lids and kept at room temperature (20–22 °C). The container containing only meat was considered the control sample. The gases (carbon dioxide CO_2_, oxygen O_2_, ammonia NH_3_, carbon monoxide CO, hydrogen sulfide H_2_S) were measured using a portable gas analyzer model GA5000 (Geotech, Jimmy Hill Way, Coventry, UK) by inserting the hose of the measuring equipment in the container. The measurements were made after 7 days to ensure the start of meat decomposition processes that lead to the release of bad odor gases such as hydrogen sulfide and ammonia. The humidity measurement of zeolites, using a thermal balance (model HC103, Mettler Toledo, Switzerland) was performed before they were placed in the meat container and 7 days after being put in the meat container. The samples of each zeolite (Cal 1, Cal 2, HCl 1, HCl 2, NaOH 1 and NaOH 2) were measured in duplicate.

Equation (1) was used to evaluate the best zeolites. Each of the five evaluation criteria has a different weight in calculating the final score obtained by each test. NH_3_ is a marker for the abundant presence of nitrogen-reducing organisms, while H_2_S is a marker for the advanced decomposition of meat products [28,29]. For the moisture level grade, the difference between the initial humidity level and the final humidity level was considered.

(1) $$FG_{zeolite}=2\times G_{\mathrm{NH}3}+2\times G_{H2S}+G_{\mathrm{CO}2}+G_{O2}+4\times G_{MC}$$

where *FG_zeolite_* is the final grade of the zeolite, which is between 10 and 100.

*G*_NH3_ is the grade for the amount of ammonia, which is between 5 and 10. The sample with the highest NH_3_ concentration will receive a score of 5 and the sample with the lowest NH_3_ concentration will receive a score of 10; *G*_H2S_ is the grade for the amount of H_2_S, which is between 5 and 10, the sample with the highest H_2_S concentration will receive a score of 5 and the sample with the lowest H_2_S concentration will receive a score of 10; *G*_CO2_ is the grade for the amount of CO_2_, which is between 5 and 10, the sample with the highest CO_2_ concentration will receive a score of 5 and the sample with the lowest CO_2_ concentration will receive a score of 10; *G*_O2_ is the grade for the amount of O_2_, which is between 5 and 10, the sample with the highest O_2_ concentration will receive a score of 10 and the sample with the lowest O_2_ concentration will receive a score of 5; *G*_MC_ is the grade for the amount of moisture, which is between 5 and 10, the sample the adsorbed the lowest humidity will receive a score of 5 and the sample the adsorbed the highest humidity will receive a score of 10.

The zeolites that had the best results were further used to evaluate their capacity in adsorbing tobacco.

Experiment 2: A glass aquarium with a volume of 54 L and dimensions 60 × 30 × 30 cm^3^ (L × W × D) was used in the study. A silicone sealed Plexiglas with a cutout of 15 × 15 cm^3^ was used as cover. A lid was made with a sealing gasket with dimensions 1.5 cm larger than the cutout. It was fixed in plexiglass with screws, so that the whole assembly can be sealed. The dimensions of the cut-out allow the easy introduction of both tobacco and zeolites. In addition, two holes were made on the filter for sampling. These were closed tightly until the time of sampling and after sampling. A sealed chamber was considered a control, and no zeolite was introduced into it. In the rest of the sealed chambers, 5 g of zeolite was introduced. Ignition gel used in HORECA field was used to maintain combustion. Then, 2.5 g of tobacco were burned inside each airtight chamber. Two samples of each zeolite (Cal 1, HCl 2, NaOH 2) were tested.

Measurements for the sample/control chamber and measurements for each type of zeolite (Table 2) were performed.

The analysis of the polycyclic aromatic hydrocarbons content at the bottom of the test chamber was also performed. That was done to demonstrate that the PAHs quantified following the analysis performed on the zeolitic material are due largely to the adsorption process and not to the deposition process on zeolite.

The content of the zeolite moisture and volatile substances was determined using a thermal balance.

### 2.6. PAHs Analysis

An extraction of both the filter and the cotton buds, as well as of the zeolites was performed with 25 mL of hexane in an ultrasonic bath for 30 min to ensure optimal extraction. After filtration, the extract was concentrated to dryness using a rotary evaporator with vacuum pump. The extract was redissolved in 1 mL of acetonitrile injected into a high-pressure liquid chromatograph with fluorescence detector HPLC-FLD to quantify the PAHs presented in Table 3.

### 2.7. Statistical Data Analysis

The Minitab 17 software (State College, PA, USA) was used to do the correlation and the surface plots for the data obtained in the experiments. 

## 3. Results and Discussion

### 3.1. Characterization of Zeolites

**Figure 1:**
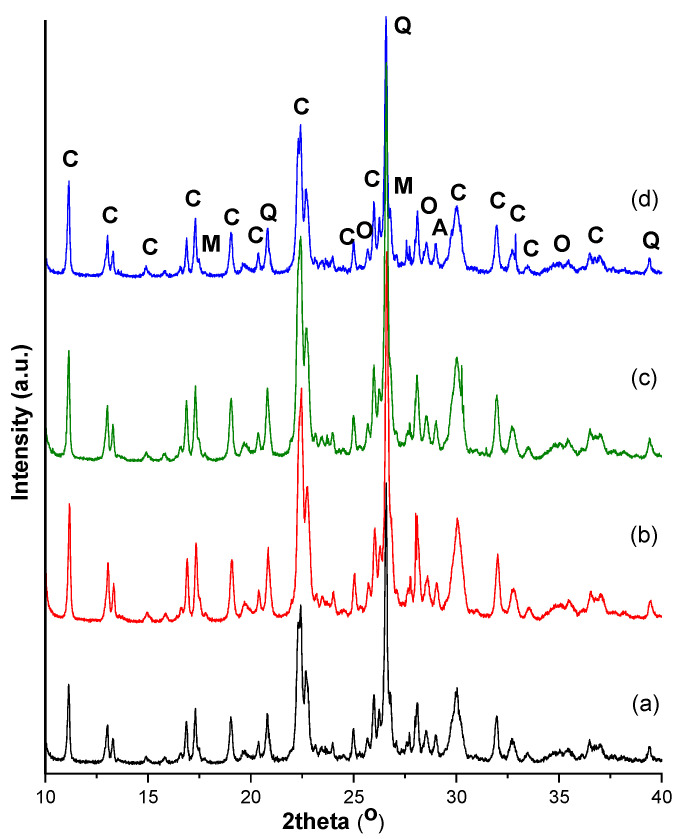
The XRD diffraction patterns of zeolite samples: (**a**) initial, (**b**) Cal 1, (**c**) HCl 1 and (**d**) NaOH 1 (Note: clinoptilolite, C; muscovite, M; quartz, Q; orthoclase, O and albite, A).

According to XRD analysis, the investigated zeolite samples contain up to 60% clinoptilolite (PDF 01-089-7538) accompanied by muscovite (PDF 00-058-2034), quartz (PDF 00-005-0490), orthoclase (PDF 01-076-0823) and albite (PDF 01-089-6423) (Figure 1). The degree of crystallinity of studied zeolites was similar, of approximately 85%. The activation treatments applied do not produce significant structural changes detectable by XRD with respect to the initial zeolite.

**Figure 2:**
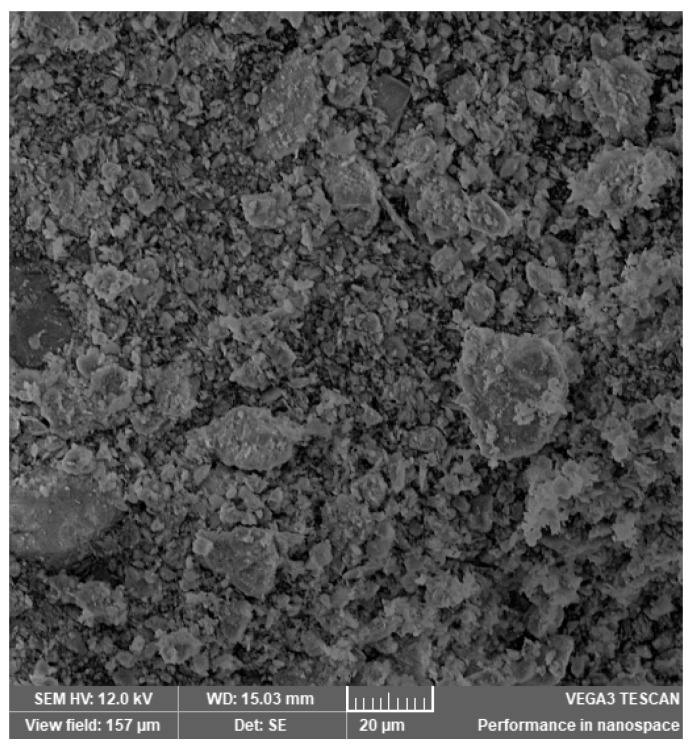
The SEM analysis of the initial zeolite sample.

**Figure 3:**
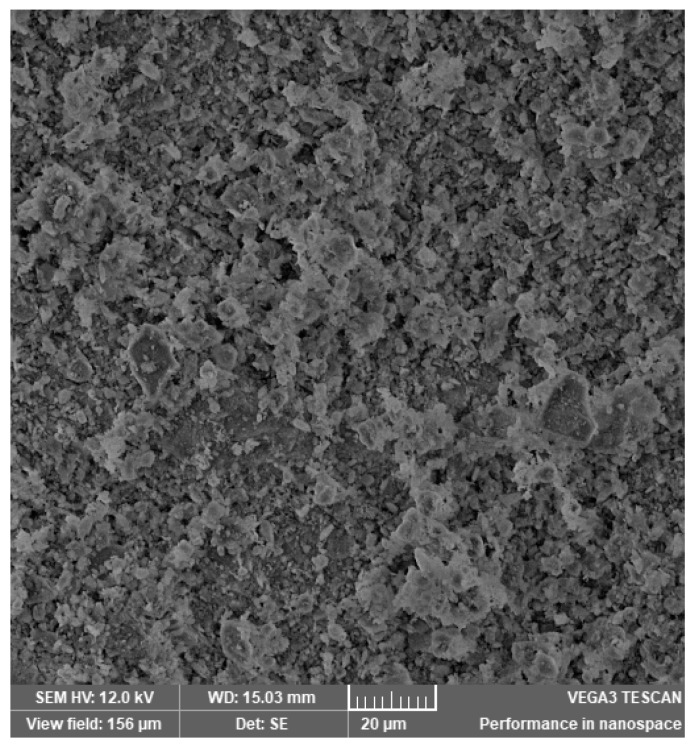
The SEM analysis of the zeolite sample Cal 1.

**Figure 4:**
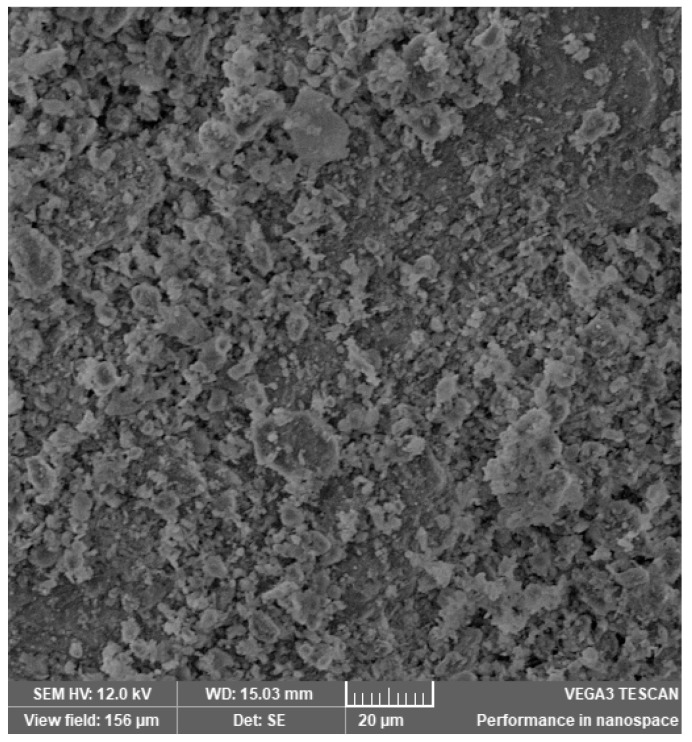
The SEM analysis of the zeolite sample Cal 2.

**Figure 5:**
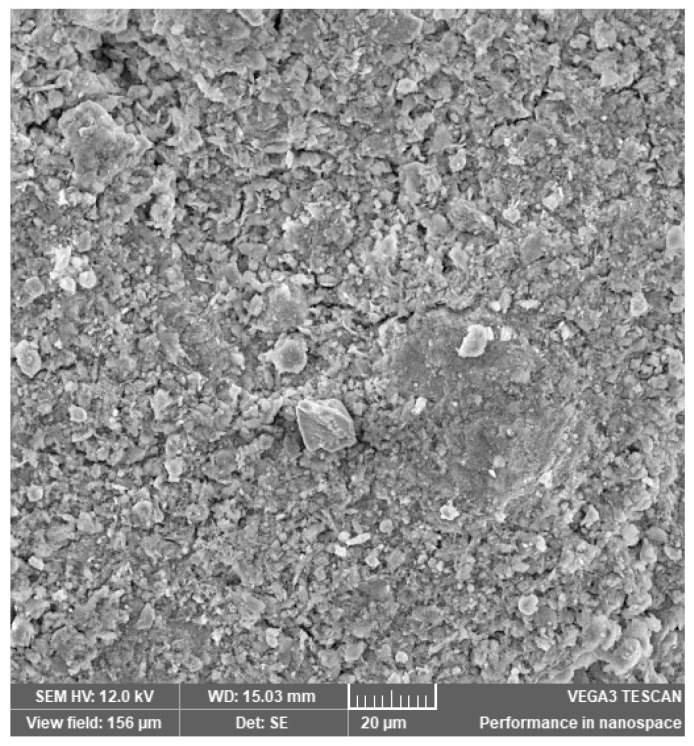
The SEM analysis of the zeolite sample NaOH 1.

**Figure 6:**
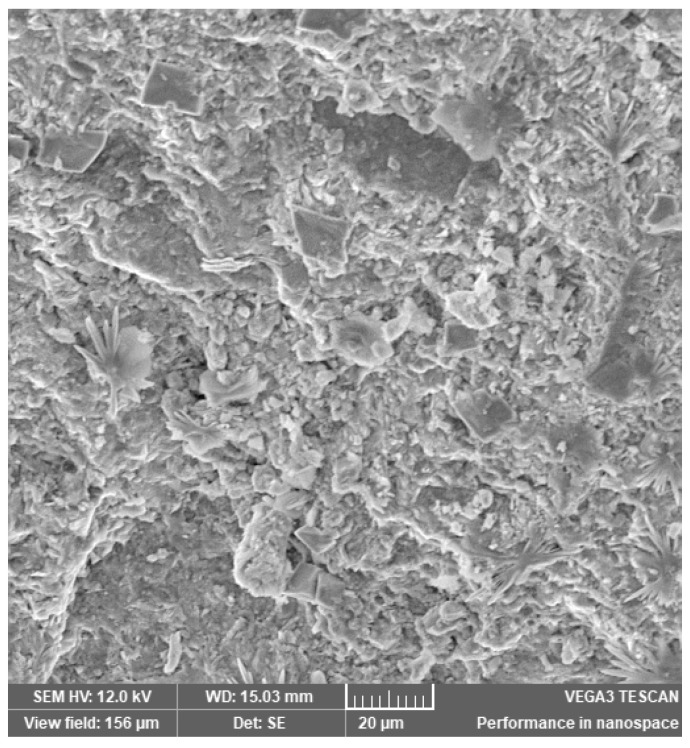
The SEM analysis of the zeolite sample NaOH 2.

**Figure 7:**
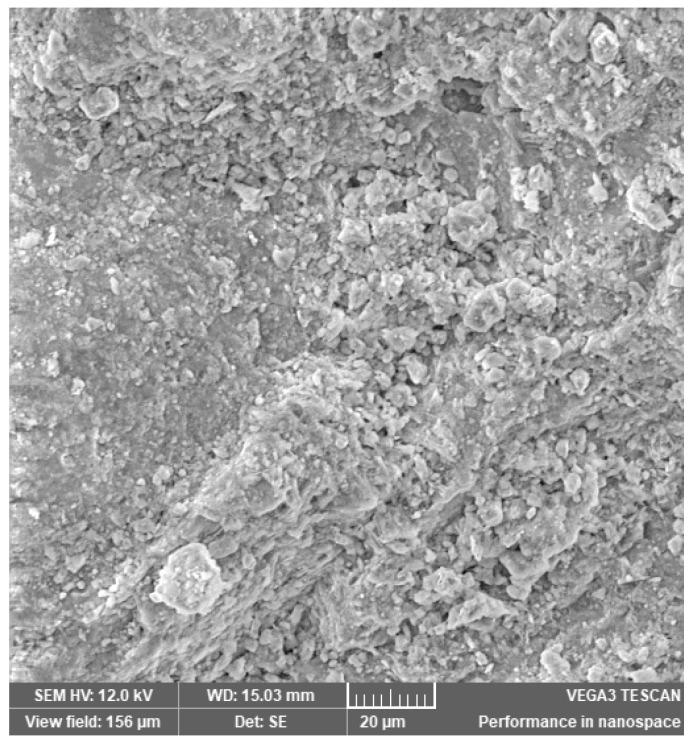
The SEM analysis of the zeolite sample HCl 1.

**Figure 8:**
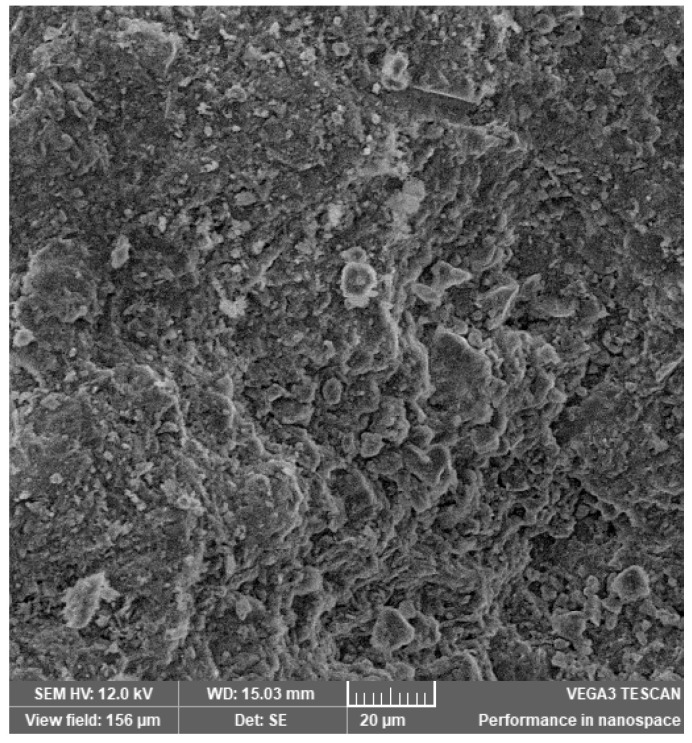
The SEM analysis of the zeolite sample HCl 1.

In Figure 2, Figure 3, Figure 4, Figure 5, Figure 6, Figure 7 and Figure 8, the images obtained from SEM for the surface structure of the zeolite are presented.

The SEM structural analysis presents a typical morphology of the sampled zeolite with irregular particles, with sharp edges, due to the different zeolite phases (crystalline and amorphous materials), in agreement with the XRD analysis. The calcined samples presented no significant differences in the SEM images. The basic and acid treatment caused grinding around the edges. The obtained results are in accordance with those reported by San Cristóbal [30] and Elaiopoulos [31].

The chemical elemental composition (wt. %) and loss of ignition (LOI) of zeolite samples is presented in Table 4. All zeolites have a Si/Al ratio over 4, which prove that they confirm the presence of clinoptilolite [32]. Zeolites that have a high Si/Al ratio are hydrophobic. [30,32]. The highest Si/Al ratio between Si and Al was obtained for the zeolite sample HCl 2 and the lowest Si/Al for the initial zeolite sample.

During the acid treatment, the pores are opened, the channels are cleaned and the isomorphic replacement of the alkali and alkaline-earth metal ions in the zeolite structure with protons (H+) takes place [32]. The oxide repartition in the sample and the LOI values are presented in Table 4. Zeolite samples with a particle size of 3–5 mm showed lower LOI values, compared to zeolite samples with a particle size of 1–3 mm, which indicates that the particle size of zeolites influences the LOI. Zeolite with a particle size of 1–3 mm had higher values for all the treatment methods.

### 3.2. Experiment 1

**Figure 9:**
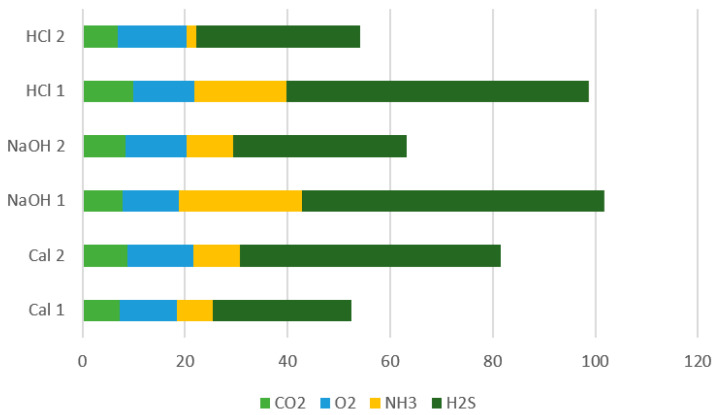
Level of adsorption of measured gases on the different zeolites.

The results obtained during the experiments for evaluating the degree of moisture adsorption are presented in Table 5 and Figure 9.

The biggest difference between the initial and the final humidity value was recorded in the calcined samples, but the highest humidity value was in the zeolite samples treated basically with NaOH.

Table 6 presents the results obtained in the tests for evaluating the degree of odor adsorption. Methane was not detected in any of the samples.

The biggest difference between the initial and the final humidity value was determined in the calcined samples, but the highest humidity value was determined in the zeolite samples basically treated with NaOH.

Ammonia and hydrogen sulfide are very important indicators in the evaluation of the degree of adsorption of unpleasant odors. These compounds are responsible for unpleasant odors due to food spoilage [28,29]. It is observed that in the analyzed air from the blank sample, the NH_3_ concentration is 37 ppm, while in the rest of the containers the NH_3_ concentration is 2–24 ppm. This proves that zeolites can adsorb NH_3_ in different concentrations, depending on the type of treatment applied for zeolites activation.

In the blank sample, the value of hydrogen sulfide content (H_2_S) is 90 ppm while in the zeolite containers its value was between 27–61 ppm. The lowest value of NH_3_ content was recorded in the sample with zeolite activated by treatment with HCl of particle sizes of 1–3 mm, 2 ppm. The lowest value of H_2_S content was recorded in the sample with calcined zeolite with particle sizes of 1–3 mm, 27 ppm.

The grades obtained by each zeolitic material are presented in Table 7.

Based on the ranking in Equation (1) the zeolites with the highest grade were Cal 1, 93, NaOH 2, 81, and HCl 2, 78. These were further used for experiment 2.

### 3.3. Experiment 2

No significant difference was recorded between the flue gases measured for each experiment, which shows that the values obtained for PAHs in the control sample can be used for comparison with the other samples in which zeolites were used.

**Figure 10:**
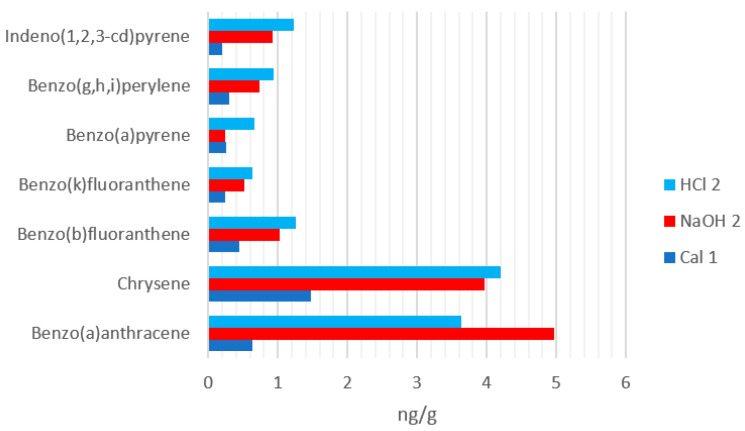
Polycyclic aromatic hydrocarbons (PAHs) adsorption on the different zeolites.

The results obtained for each type of sample analyzed in terms of PAHs content are presented in Table 8, Table 9 and Table 10 and Figure 10.

In the control sample, the amount of PAHs deposited on the wall is higher than in the samples with zeolites, 20.74 ng/cm^2^ compared to 2.73–3.38 ng/cm^2^ in the samples with zeolites. The large difference between the amount at the bottom of the chamber in which zeolites were not introduced and the amount in which zeolites were introduced is due to the capacity and degree of adsorption of PAHs by zeolites. This demonstrates that natural zeolites can adsorb PAHs and thus they can purify the air of cigarette smoke.

There is a significant difference between the amount of PAHs quantified on the PM10 filter in the control sample compared to the samples in which zeolites with different characteristics were introduced, 75.69 ng/m^3^ compared to 1.82–3.42 ng/m^3^. The zeolite with particle sizes of 3–5 mm activated by acid treatment adsorbed the highest amount of PAHs, namely 89.56 ng/g. The smallest amount of PAH was adsorbed by zeolite with particle sizes of 1–3 mm, activated by calcination, 38.92 ng/g. The zeolite using NaOH and particle sizes 3–5 mm adsorbed 65.56 ng/g, a result which is consistent with the finding of Buchori, Araújo and Wirawan [33,34,35], namely that the interaction of π-electrons in the PAH (i.e., van der Waals forces) and the hydrophobicity of the zeolite ensure a bigger adsorption capacity. In Table 11 the correlation between the different measured parameters is presented for samples Cal1, NaOH 2 and HCl 2.

There is a negative correlation of –0.999 between the Si/Al and the humidity adsorbed. Between the Si/Al and the PAHs adsorption there is a positive correlation of 0.976. There is no significant correlation between Si/Al and the H_2_S adsorbed.

**Figure 11:**
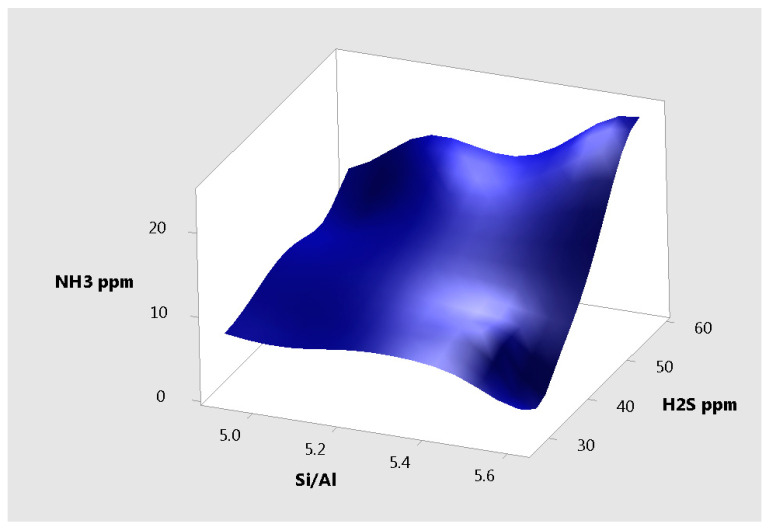
Surface plot of NH_3_ vs. Si/Al, H_2_S.

The surface plot for NH_3_ vs. Si/Al, H_2_S (Figure 11) for all the samples from experiment 1 further illustrate the low correlation of Si/Al to the measured parameters.

**Figure 12:**
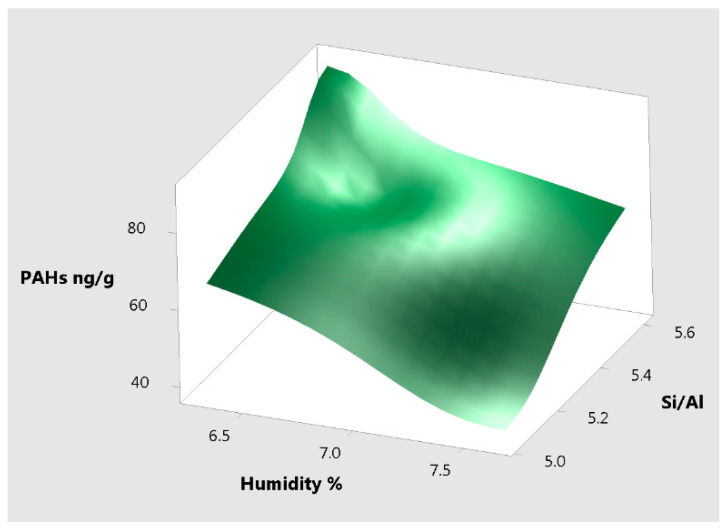
Surface plot of PAHs vs. Si/Al, humidity.

Figure 12 shows the surface plot of PAHs vs. Si/Al, humidity for samples Cal1, NaOH 2 and HCl 2 which illustrates the positive and negative correlation of these measured parameters.

## 4. Conclusions

This study confirms that natural zeolites are low-cost materials for odor control and removal. The thermal and chemical treatments greatly influence the zeolites capacity of adsorption. While the thermally-activated zeolite had a significantly better performance regarding humidity control, the acid treated zeolite had the best results in adsorbing the PAHs from the atmosphere. The zeolite with particle sizes of 3–5 mm activated by acid treatment adsorbed twice as much PAHs (89.56 ng/g) from air as the zeolite that was thermally treated (38.92 ng/g). The difference is even bigger when it comes to PAHs with a higher number of aromatic rings. The HCl 2 sample adsorbed 0.66 ng/g benzo(a)pyrene, while the Cal1 adsorbed only 0.25 ng/g.

The activation treatment applied to the different natural zeolites has a great influence on adsorption specificity and capacity. Different activation treatments offer the possibility to make tailored natural zeolites for different applications. Further studies must be done on a mixture of natural zeolites with other adsorbent materials to create an even better tailored product.

## Figures and Tables

**Table 1:** Tested zeolite samples.

Sample Code	Description
Initial 1	Zeolite, particle size 1–3 mm
Initial 2	Zeolite, particle size 3–5 mm
Cal 1	Thermally treated zeolite, particle size 1–3 mm
Cal 2	Thermally treated zeolite, particle size 3–5 mm
HCl 1	Chemically modified zeolite with HCl, particle size 1–3 mm
HCl 2	Chemically modified zeolite with HCl, particle size 3–5 mm
NaOH 1	Chemically modified zeolite with NaOH, particle size 1–3 mm
NaOH 2	Chemically modified zeolite with NaOH, particle size 3–5 mm

**Table 2:** Experiments performed for evaluation of the degree of adsorption of tobacco smell.

Test Type	Performed Analysis	Burning Interval
Air from the sealed chamber into which only tobacco was introduced (control sample)	Fuel gases	Immediately after the combustion stopped
Sampling on PM10 particle filter from the sealed chamber in which only tobacco was introduced (control sample)	Polycyclic aromatic hydrocarbons	Immediately after the combustion stopped
Air from the sealed chamber	Fuel gases	Immediately after the combustion stopped
Cal 1, NaOH, 2 HCl 2	Polycyclic aromatic hydrocarbons	24 h after the combustion stopped
Sampling with cotton buds from the bottom of the sealed chamber	Polycyclic aromatic hydrocarbons	24 h after the combustion stopped
Sampling on PM10 particle filter from the sealed chamber	Polycyclic aromatic hydrocarbons	24 h after the combustion stopped

**Table 3:** Stages and parameters of the PAHs determination method.

Step	Equipment/Materials Used	Parameters
Extraction	Ultrasonic bath, LBS, Falc, Italy	Extraction solvent: 25 mL Hexane Duration: 30 min Temperature: 20–22 °C
Purification	Cellulose filter without metals	sup>∙ Diameter 150 mm
Concentration	Rotavapor HeiVap Precision Heidolph, Schwabach, Germany	Vacuum: 200 mbar Temperature: 40 °C
Analysis	HPLC-FLD, Perkin Elmer 200 Series Shelton, CT, USA	Mobile phase: Ultrapure water and acetonitrile in gradient program Flow rate: 0.7 mL/min Injection volume: 50 µL Chromatographic column: Intersil HPLC Column ODS-P 5µ 4.6 × 150 mm GL Science Specific wavelength program Duration: 45 min

**Table 4:** Chemical composition (wt.%) of zeolite samples.

Sample	Initial 1	Initial 2	Cal 1	Cal 2	NaOH 1	NaOH 2	HCl 1	HCl 2
**Na_2_O**	0.33 ± 0.03	0.33 ± 0.02	0.44 ± 0.03	0.45 ± 0.03	3.40 ± 0.31	3.45 ± 0.32	<0.14	<0.14
**K_2_O**	2.87 ± 0.24	2.47 ± 0.30	2.47 ± 0.34	3.01 ± 0.27	2.41 ± 0.24	1.87 ± 0.20	3.10 ± 0.29	2.34 ± 0.26
**CaO**	2.48 ± 0.29	2.47 ± 0.33	3.19 ± 0.27	2.60 ± 0.29	4.83 ± 0.39	5.24 ± 0.56	1.72 ± 0.18	2.16 ± 0.24
**MgO**	<0.14	0.96 ± 0.09	0.97 ± 0.12	1.17 ± 0.11	0.83 ± 0.09	0.98 ± 0.08	1.02 ± 0.11	1.10 ± 0.13
**Al_2_O_3_**	12.64 ± 1.23	12.64 ± 1.29	12.33 ± 1.30	12.57 ± 1.28	10.58 ± 1.29	10.96 ± 1.10	12.41 ± 1.21	11.64 ± 1.21
**Fe_2_O_3_**	2.54 ± 0.19	2.55 ± 0.21	2.31 ± 0.26	2.53 ± 0.19	1.91 ± 0.23	1.93 ± 0.17	2.27 ± 0.20	2.20 ± 0.17
**SiO_2_**	69.79 ± 7.04	69.79 ± 6.78	70.50 ± 7.06	70.22 ± 7.11	67.24 ± 6.83	67.78 ± 6.67	72.38 ± 7.29	74.04 ± 7.33
**LOI**	4.87 ± 0.45	4.87 ± 0.42	5.04 ± 0.49	4.92 ± 0.51	5.60 ± 0.48	5.45 ± 0.56	5.14 ± 0.49	5.61 ± 0.55

**Table 5:** Levels of zeolite humidity adsorption.

Sample	Humidity, Initial (%)	Humidity, Final (%)
Cal 1	0.62 ± 0.03	8.26 ± 0.41
Cal 2	0.30 ± 0.02	6.65 ± 0.33
NaOH 1	3.27 ± 0.16	9.72 ± 0.49
NaOH 2	2.61 ± 0.13	9.28 ± 0.46
HCl 1	2.55 ± 0.13	8.85 ± 0.44
HCl 2	3.16 ± 0.16	8.44 ± 0.42

**Table 6:** Results of experiment 1.

Parameter	Control	Cal 1	Cal 2	NaOH 1	NaOH 2	HCl 1	HCl 2
CO_2_ (%)	12.6 ± 1.4	7.2 ± 0.8	8.8 ± 1.0	7.8 ± 0.9	8.3 ± 0.9	9.9 ± 1.1	6.9 ± 0.8
O_2_ (%)	8 ± 0.9	11.2 ± 1.2	12.8 ± 1.4	10.9 ± 1.2	12 ± 1.3	11.9 ± 1.3	13.3 ± 1.5
NH_3_ (ppm)	37 ± 4.1	7 ± 0.8	9 ± 1.0	24 ± 2.6	9 ± 1.0	18 ± 2.0	2 ± 0.2
CO (ppm)	9 ± 1.0	6 ± 0.7	2 ± 0.2	7 ± 0.8	5 ± 0.6	6 ± 0.7	4 ± 0.4
H_2_S (ppm)	90 ± 9.9	27 ± 3.0	51 ± 5.6	59 ± 6.5	34 ± 3.7	59 ± 6.5	32 ± 3.5

**Table 7:** Grade obtained by each zeolite sample and the final grade base on Equation (1).

Sample	CO_2_	O_2_	NH_3_	H_2_S	Humidity	Final Grade
Cal 1	9	6	9	10	10	93
Cal 2	6	9	8	7	7	73
NaOH 1	8	5	5	5	8	65
NaOH 2	7	8	7	8	9	81
HCl 1	5	7	6	6	6	60
HCl 2	10	10	10	9	5	78

**Table 8:** Results for samples taken on cotton buds.

Parameter	Obtained Value (ng/m^3^)			
	Blank	Cal 1	NaOH 2	HCl 2
Naphthalene	0.88 ± 0.11	<LQ *	<LQ *	<LQ *
Acenaphthene	0.15 ± 0.02	<LQ *	<LQ *	<LQ *
Fluorene	0.72 ± 0.09	0.72 ± 0.09	0.15 ± 0.02	0.55 ± 0.07
Phenanthrene	0.72 ± 0.09	0.49 ± 0.06	0.97 ± 0.12	0.75 ± 0.09
Anthracene	0.87 ± 0.10	0.05 ± 0.01	0.41 ± 0.05	0.49 ± 0.06
Fluoranthene	2.13 ± 0.26	0.59 ± 0.07	0.67 ± 0.08	0.51 ± 0.06
Pyrene	5.68 ± 0.68	0.52 ± 0.06	0.64 ± 0.08	0.32 ± 0.04
Benzo(a)anthracene	8.68 ± 1.04	0.19 ± 0.02	0.32 ± 0.04	0.21 ± 0.03
Chrysene	0.92 ± 0.11	0.17 ± 0.02	0.22 ± 0.03	0.12 ± 0.01
Benzo(b)fluoranthene	<LQ *	<LQ *	<LQ *	<LQ *
Benzo(k)fluoranthene	<LQ *	<LQ *	<LQ *	<LQ *
Benzo(a)pyrene	<LQ *	<LQ *	<LQ *	<LQ *
Dibenzo(a,h)anthracene	<LQ *	<LQ *	<LQ *	<LQ *
Benzo(g,h,i)perylene	<LQ *	<LQ *	<LQ *	<LQ *
Indeno(1,2,3-cd)pyrene	<LQ *	<LQ *	<LQ *	<LQ *
Total PAHs	20.74 ± 2.49	2.73 ± 0.33	3.38 ± 0.41	2.95 ± 0.35

* LQ = 0.05 ng/m^3^.

**Table 9:** Results for samples taken on PM10 particulate filter.

Parameter	Obtained Value (ng/m^3^)			
	Blank	Cal 1	NaOH 2	HCl 2
Naphthalene	11.36 ± 1.36	0.31 ± 0.04	2.09 ± 0.25	2.30 ± 0.28
Acenaphthene	0.81 ± 0.10	<LQ *	<LQ *	<LQ *
Fluorene	11.60 ± 1.39	0.63 ± 0.08	0.49 ± 0.06	0.24 ± 0.03
Phenanthrene	32.13 ± 3.86	0.63 ± 0.08	0.50 ± 0.06	0.19 ± 0.02
Anthracene	3.03 ± 0.36	0.03 ± 0.00	<LQ *	<LQ *
Fluoranthene	9.19 ± 1.10	0.13 ± 0.02	0.18 ± 0.02	0.18 ± 0.02
Pyrene	0.77 ± 0.09	0.09 ± 0.01	0.17 ± 0.02	0.17 ± 0.02
Benzo(a)anthracene	1.11 ± 0.13	<LQ *	<LQ *	<LQ *
Chrysene	4.32 ± 0.52	<LQ *	<LQ *	<LQ *
Benzo(b)fluoranthene	0.36 ± 0.04	<LQ *	<LQ *	<LQ *
Benzo(k)fluoranthene	0.12 ± 0.01	<LQ *	<LQ *	<LQ *
Benzo(a)pyrene	0.15 ± 0.02	<LQ *	<LQ *	<LQ *
Dibenzo(a,h)anthracene	<LQ	<LQ *	<LQ *	<LQ *
Benzo(g,h,i)perylene	0.12 ± 0.01	<LQ *	<LQ *	<LQ *
Indeno(1,2,3-cd)pyrene	0.59 ± 0.07	<LQ *	<LQ *	<LQ *
Total PAHs	75.69 ± 9.08	1.82 ± 0.22	3.42 ± 0.41	3.08 ± 0.37

* LQ = 0.05 ng/m^3.^

**Table 10:** Results for the degree of PAHs by zeolites adsorption.

Parameter	PAHs (ng/g)		
	Cal 1	NaOH 2	HCl 2
Naphthalene	0.32 ± 0.04	0.52 ± 0.06	0.91 ± 0.11
Acenaphthene	0.31 ± 0.04	0.26 ± 0.03	0.14 ± 0.02
Fluorene	0.55 ± 0.07	4.31 ± 0.52	8.98 ± 1.08
Phenanthrene	12.67 ± 1.52	15.37 ± 1.84	22.69 ± 2.72
Anthracene	3.16 ± 0.38	2.97 ± 0.36	1.53 ± 0.18
Fluoranthene	9.10 ± 1.09	14.67 ± 1.76	21.94 ± 2.63
Pyrene	9.26 ± 1.11	15.06 ± 1.81	20.67 ± 2.48
Benzo(a)anthracene	0.64 ± 0.08	4.97 ± 0.60	3.63 ± 0.44
Chrysene	1.47 ± 0.18	3.97 ± 0.48	4.20 ± 0.50
Benzo(b)fluoranthene	0.45 ± 0.05	1.03 ± 0.12	1.26 ± 0.15
Benzo(k)fluoranthene	0.24 ± 0.03	0.52 ± 0.06	0.63 ± 0.08
Benzo(a)pyrene	0.25 ± 0.03	0.24 ± 0.03	0.66 ± 0.08
Dibenzo(a,h)anthracene	<LQ *	<LQ *	0.16 ± 0.02
Benzo(g,h,i)perylene	0.30 ± 0.04	0.74 ± 0.09	0.94 ± 0.11
Indeno(1,2,3-cd)pyrene	0.20 ± 0.02	0.93 ± 0.11	1.23 ± 0.15
Total PAHs	38.92 ± 4.67	65.56 ± 7.87	89.56 ± 10.75

* LQ = 0.05 ng/g.

**Table 11:** Correlation between the different measured parameters.

Parameter	NH_3_	H_2_S	Humidity	Si/Al	PAHs
**NH_3_**	1.000	-	-	-	-
**H_2_S**	0.038	1.000	-	-	-
**Humidity**	0.491	−0.852	1.000	-	-
**Si/Al**	−0.495	0.849	−1.000	1.000	-
**PAHs**	−0.671	0.715	−0.975	0.976	1.000

## Data Availability

Data sharing is not applicable.
